# An efficient protein complex mining algorithm based on Multistage Kernel Extension

**DOI:** 10.1186/1471-2105-15-S12-S7

**Published:** 2014-11-06

**Authors:** Xianjun Shen, Yanli Zhao, Yanan Li, Tingting He, Jincai Yang, Xiaohua Hu

**Affiliations:** 1 School of Computer, Central China Normal University, Wuhan, 430079, China; 2 College of Computing and Informatics, Drexel University, Philadelphia, PA, USA

**Keywords:** protein complexes, protein-protein interaction network, multistage kernel extension

## Abstract

**Background:**

In recent years, many protein complex mining algorithms, such as classical clique percolation (CPM) method and markov clustering (MCL) algorithm, have developed for protein-protein interaction network. However, most of the available algorithms primarily concentrate on mining dense protein subgraphs as protein complexes, failing to take into account the inherent organizational structure within protein complexes. Thus, there is a critical need to study the possibility of mining protein complexes using the topological information hidden in edges. Moreover, the recent massive experimental analyses reveal that protein complexes have their own intrinsic organization.

**Methods:**

Inspired by the formation process of cliques of the complex social network and the centrality-lethality rule, we propose a new protein complex mining algorithm called Multistage Kernel Extension (MKE) algorithm, integrating the idea of critical proteins recognition in the Protein- Protein Interaction (PPI) network,. MKE first recognizes the nodes with high degree as the first level kernel of protein complex, and then adds the weighted best neighbour node of the first level kernel into the current kernel to form the second level kernel of the protein complex. This process is repeated, extending the current kernel to form protein complex. In the end, overlapped protein complexes are merged to form the final protein complex set.

**Results:**

Here MKE has better accuracy compared with the classical clique percolation method and markov clustering algorithm. MKE also performs better than the classical clique percolation method both on Gene Ontology semantic similarity and co-localization enrichment and can effectively identify protein complexes with biological significance in the PPI network.

## Introduction

Mining protein complexes is very important in biological processes since it helps reveal the structure-functionality relationships in biological networks. So much attention has been paid to accurate detection of protein complexes from the increasing amount of protein-protein interaction (PPI) network data. In recent years, many protein complex mining algorithms have developed for protein-protein interaction network. However, most of the available algorithms primarily concentrate on mining dense protein subgraphs as protein complexes, failing to take into account the inherent organizational structure within protein complexes. Thus, there is a critical need to study the possibility of mining protein complexes using the topological information hidden in edges [1]. Moreover, the recent massive experimental analyses reveal that protein complexes have their own intrinsic organization [2].

Complex social networks and complex biological networks both contain distinct community structures [3]. The formation of complex social network is often divided into several stages. First, the founders create the original kernel of the community according to common ideas or interests. Next, the kernel community is expanded by introducing the similar objects to join the community to form a basic framework and organizational structure, and the new community begins to run effectively. Subsequently, the community gradually assimilates objects sharing common ideas or interests incessantly joining this community, then a complex network is constructed which exerts corresponding influence and function in society.

Studies show that the PPI network is such a kind of the complex network, it has the properties similar to complex networks on topological structure, namely small world property [4], scale-free property [5], and it presents remarkable modular structures [6]. The PPI network is composed by many protein complexes (function modules or clusters), and these protein complexes are made up of some proteins working together to carry out some functions. Protein complex refers to a group of proteins that interact with each other at the same time and in the same space. The formation of the protein complexes and the PPI follow its inherent objective laws, which is a gradually developing process, not accomplished at one stroke.

Halt et al. believed that criticality is an important property of protein complexes, and experimental data shows that critical proteins always heavily concentrate in certain complexes [7]. Some researchers combined the recognition of the critical proteins with protein complexes detection. Zotenko et al. pointed out that densely connected protein complexes with same or similar biological function are rich in critical protein nodes, and these nodes around the critical nodes have a strikingly functional similarity [8]. Jeong et al. discovered the centrality-lethality rule which demonstrates that the deletion of proteins with more neighbouring nodes is easier to affect the topological structure of the whole network, and then produces lethal effect on the body [9]. That is to say, the protein nodes with higher degree more tend to exhibit the criticality in biological properties and play an important role in the protein complexes.

Based on the above ideas, we propose a novel protein complex mining algorithm called MKE (Multistage Kernel Extension) based on multistage kernel extension. MKE first transforms the undirected and unweighted graph of PPI network to a directed and weighted network graph, then selects the node set composed by high-degree and closely connected nodes in PPI network as the first level kernel of the protein complex, or as the kernel nodes of the protein complex, since these nodes are prone to play a key role in the biological function of the protein complex. Next, for each adjacent node of the first level kernel of the protein complex, MKE uses the definition of the weighted best neighbour node to determine the extent of the closeness between the adjacent node and the current kernel. If the extent of the closeness is greater than the average extent of closeness of the subgraph formed by the current kernel and its neighbouring nodes, then this neighbouring node can be added into the current kernel and be extended into the next level kernel. This process is continuously repeated; the kernel is extended stage-by-stage and finally a protein complex is constructed. Experimental results demonstrate that MKE is simple and effective, and the protein complexes identified with biological significance have a very high degree of match with reference protein complexes.

## Methods

### Constructing directed and weighted network graph

In the protein-protein interaction network, for each pair of protein nodes, it is difficult to determine whether they belong to the same protein complex just by the degree of the nodes and their connection characteristics. Since two protein nodes have their own neighbour node set in PPI network, we can get their common neighbour node set. If one pair of protein nodes has more common neighbour nodes, it indicates that they have closer connection. Thus the possibility that the two proteins belong to the same protein complex is greater, and the probability that they participate in the same cell function is larger as well. Therefore, the common neighbour node set of one pair of protein nodes acts as an important role in weighing the relationship between the pair of protein nodes.

In the PPI network, for any two protein nodes, denoted by *s * and *t *, if there is an undirected edge between them, it can be converted into directed and weighted edge. Initially, the edge between node *s * and node *t * is undirected and unweighted.

$N_{s}$ and $N_{t}$ represent the neighbour node set of node *s * and node *t * respectively in the PPI network, and the number of the common neighbour nodes between them is denoted by $cn_{st}$, defined by:

(1) $$cn_{st}=\left|N_{s}\cap N_{t}\right|$$

$w_{st}$ and $w_{ts}$ are the directed and weighted edges between node *s * and node *t * after conversion treatment, which is described in (2) and (3):

(2) $$w_{st}=cn_{st}/d_{s}$$

(3) $$w_{ts}=cn_{ts}/d_{t}$$

**Figure 1:**
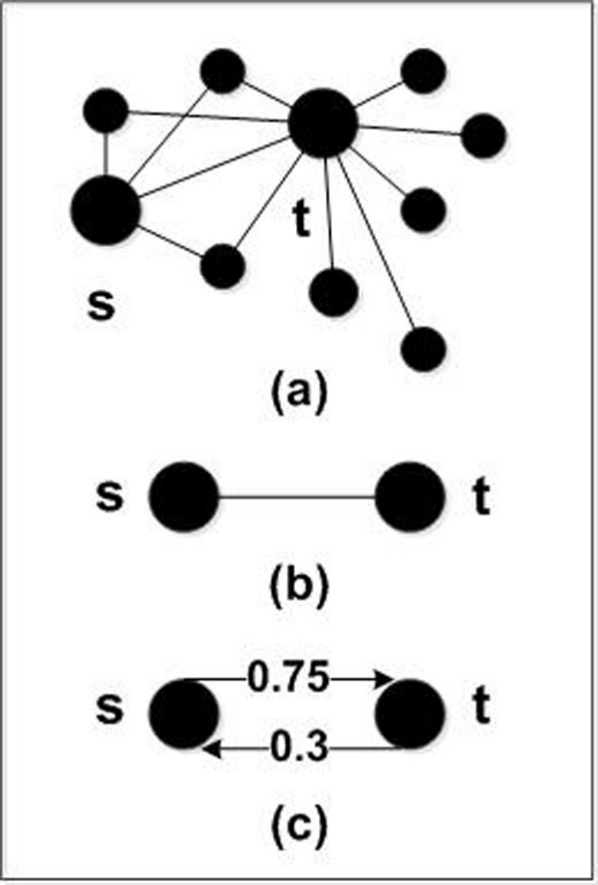
**Convert the undirected edge to directed and weighted edges**. (a) is an interactive graph (undirected and unweighted graph) including node *s * and node *t *.(b) shows the undirected and unweighted edge between node *s * and node *t *. (c) shows the directed and weighted edges between node *s * and node *t * after conversion treatment.

Where, $cn_{st}$ is the number of the common nodes between node *s * and node *t *, $cn_{ts}$ is the number of common nodes between node *t * and node *s *. Since it is an undirected graph, $cn_{st}=cn_{ts}$. $d_{s}$ represents the degree of node *s *, and $d_{t}$ represents the degree of node *t *. After the conversion treatment, there are two directed and weighted edges between the node *s * and node *t *, as shown in Figure 1.

In the above definitions, the weights of the edges between two nodes are unequal. Assume that the degree of protein node *t * is very large, while the degree of protein node *s * is so small. Although they have the same number of common neighbour nodes, according to formula (2) and (3), $w_{st}$ >$w_{ts}$, as shown in Figure 1. In terms of node *t *, the possibility that node *t * and node *s * belong to the same protein complex is small, but in terms of node *s *, the possibility that node *s * and node *t * belong to the same protein complex is larger. Since the correlation is asymmetric, it is necessary to use two directed and weighted edges to weigh the correlation between the nodes. However, in these known protein complex mining algorithms, most of them fail to consider this detail, but treat it in the way with an equal weight, which is obviously unreasonable.

If there are *n * nodes in the network, let $d_{\text{max}}$ denote the biggest degree of a node in current network. For each node *v *, find its neighbour node set *S *. For any node *t * in set *S *, the number of the edges between node *t * and the other nodes in set *S * is the number of common neighbour nodes between node *v * and node *t *, and the time complexity is $O\left(d_{\text{max}}∙d_{\text{max}}\right)$ that discovers the common neighbours. Then the time complexity is $O\left(n∙d_{\text{max}}∙d_{\text{max}}\right)$ that converts the undirected and unweighted graph into the directed and weighted network graph.

### Defining the weighted best neighbour node

In the PPI network, for one protein node, there are always a large number of neighbour nodes around it. Since the PPI graph has been transformed into a directed and weighted graph, it is convenient to find the neighbour node.

In terms of one node *v *, if the directed weights between node *v * and one of its neighbour node both greater than $w_{ave}$, then we call this neighbour node the weighted best neighbour node of node *v *, denoted by Bn(v). That is to say, the weighted best neighbour node may be more than one. The relationship of node *v * and its best neighbour node Bn(v) is shown in (4):

(4) $$\left\{\begin{array}{c} w_{v,Bn\left(v\right)}\geq w_{ave}\\ w_{Bn\left(v\right),v}\geq w_{ave} \end{array}\right)$$

Where,$w_{ave}$ is a weight threshold--an average weight of a local network formed by the current subgraph and the adjacent nodes of the subgraph. Its definition is given in the back (see formula (7)). (In the initial formation stage of the protein complex, as the initial nodes are the critical nodes with high degree and more adjacent nodes, and combining the experimental tests in this paper, the $w_{ave}$ is initialized to 0.8.)

In terms of a cluster, the neighbour nodes of the cluster refer to the nodes which have the direct interactions with the internal nodes of the cluster but are not in the cluster. For cluster *c *, $N_{c}$ is the neighbour node set of cluster *c *, *v *is one of the nodes in set $N_{c}$. *u * is the node within cluster *c *. $w_{uv}$is the weight between the node *u * within cluster *c * and its neighbour node *v *. The best neighbour node of cluster *c * can be denoted by Bn(c), and $w_{c,Bn\left(c\right)}$is the weight between cluster *c * and its best neighbour node. The relationship of cluster *c * and its best neighbour node Bn(c) is shown in (5):

(5) $$\left\{\begin{array}{c} w_{c,Bn\left(c\right)}=w_{uv}\geq w_{ave}\\ w_{Bn\left(c\right),c}=w_{vu}\geq w_{ave} \end{array}\right)\left(u\in c,v\in N_{c}\right)$$

### Identifying the first level kernel of protein complex

In the PPI network, protein complex generally corresponds to the subgraph where proteins interact closely. The formation of protein complex is a slow procedure. Compared to other part of the protein complex, the first level kernel of the protein complex corresponds to the set of kernel nodes of the protein complex, which plays an irreplaceable role in the protein complex. Since the degree of the nodes in the PPI network follows the power-law distribution, there are a few nodes with a high degree and they play an important role in the network, corresponding to the core parts of the network. Since the closely connected and high-degree nodes have no significant difference between them, they are together taken as the first level kernel of the protein complex. P(k) is the percentage that the high-degree nodes account for of all the nodes in the PPI network.

(6) $$P\left(k\right)=\frac{n_{d_{v}\geq k}}{n}$$

Where *n *denotes the number of nodes in the network, *k *is the given degree threshold, $d_{v}$represents the degree of node *v *. $n_{d_{v}\geq k}$is the total number of nodes whose degrees are greater than or equal to *k * in the PPI network. According to the different sizes of the PPI network, P(k)can be tuned properly, and in this paper it is set to be 0.01, because critical nodes account for small fraction of all nodes.

According to the degree distribution of nodes in the PPI network, first, all the nodes in the network are ranked according to the degree, and nodes with degree greater than or equal to *k * are selected to be the initial kernel nodes of the first level kernels of protein complexes. For the protein nodes within the first level kernel of the protein complex, there are plenty of common neighbour nodes between them, otherwise there is little chance that these nodes would belong to the same protein complex. Thus for two protein nodes, node *s * and node *t *, when the directed weights between them are both greater than the given threshold value of the weight, it indicates that they closely connect with each other. Then they can be thought to belong to the same first level kernel of protein complex. [see Additional file 1 for Algorithm 1--Identifying the First level Kernel(IFLK) Algorithm and for its Time Complexity Analysis].

### Identifying the second level kernel of protein complex

For each neighbour node of the identified first level kernel, we can adopt the definition of weighted best neighbour node to analyze the extent of closeness between the current kernel and the neighbour nodes. If the extent of closeness is greater than the average extent of closeness of the subgraph formed by the current kernel and its neighbour nodes, then add this neighbour node into the current kernel to generate the next kernel, otherwise, discard it.

The first level kernel of the protein complex identified is usually the single protein node with high degree or the set of some protein nodes with high degree. Compared with the first level kernel of the protein complex, the degree of the nodes of the second level kernel is slightly small. However, like the first level kernel of the protein complex, the second level kernel of the protein complex is in the central position of the protein complex as well, so there is no substantial difference on the extent of connection closeness between the second level kernel and the first level kernel of the protein complex.

Since the second level kernel of the protein complex is in the periphery of the first level kernel, we can naturally achieve the second level kernel by extending the first level kernel of the protein complex. In the protein-protein interaction network graph, the second level kernel of the protein complex can intuitively correspond to the network subgraph formed by the first level kernel and its adjacent nodes. Simply speaking, some special processing can be done on the adjacent network of the first level kernel of the protein complex, and then we can conveniently obtain the second level kernel of the protein complex.

Generally speaking, in the local area, if the number of exchanges between two people or the number of common friends of two people exceeds the average number of exchanges or the average number of common friends between people in the district, then the relationship between the two people can be regarded as unusual. Likewise, the protein complexes in the PPI network possess the same property and law. Therefore, the average weight of the local network is taken as the criterion to measure whether two nodes belong to the identical protein complex. In terms of the directed and weighted PPI graph G(V,E), the average weight $w_{ave}$ of the network can be shown as follows:

(7) $$w_{ave}=\frac{\underset{s\neq t\in V}{\sum }w_{st}}{|V|\times \left(|V|-1\right)}$$

Where, |*V*| is the number of the nodes of the network formed by the current kernel and its neighbours, $w_{st}$is the directed weight between node *s * and node *t *. [see Additional file 1 for Algorithm 2--Identifying the Second Level Kernel(ISLK) Algorithm and for its Time Complexity Analysis].

### Mining protein complexes by multistage kernel extension

In the PPI network, the protein complex is a striking module structure which is formed by multistage kernel extension of the kernel protein nodes. The initial stage of kernel extension of the protein complex is very important, so we have elaborated on this before. Since the kernel extension stages of the protein complex are similar and the next stages of kernel extension are similar to the second stage, it is redundant and pointless to elaborate on the next stages of kernel extension of the protein complex.

During the multistage kernel extension process, the previous extension stage is more important than the next extension stage. In general, the more important protein node sets account for smaller percentage in PPI network. Thereby, we can make a reasonable hypothesis accordingly that the number of the nodes added into the current protein complex kernel in the next stage is greater than that in the previous stage. In addition, due to the specificity of different networks, some kernels need to early terminate the extension to form the protein complexes after several extension stages; consequently, the algorithm introduces the Extended Level Parameter *α * as a constraint.

According to the above discussion, we implement the same process on the second level kernel like the way that the first level kernel extends to yield the second level kernel of the protein complex, and the process is repeated until the increased number $\Delta N_{current}$ of nodes of current kernel extension is smaller than the increased number $\Delta N_{prior}$ of nodes of the previous kernel extension or until the extended level parameter *α * is greater than the threshold $T_{\alpha }$, then output the ultimate kernel of the protein complex. Before the algorithm starts, the current kernel of the protein complex is null, naturally, the size of the kernel is 0. After having identified the first level kernel of protein complex, the increased number of nodes of first level kernel of the protein complex is obviously equal to its own size.

In terms of a node in the PPI network, the extent of closeness between the node and a protein complex is obtained by calculating the extent of closeness between this node and the nodes satisfying special condition within the protein complex, rather than by calculating the extent of closeness between this node and multiple nodes within the protein complex. Therefore, using the definition of the weighted best neighbour node for the PPI network, we can find that algorithm in this paper predicts the protein complex by one node in the kernel extending to the nodes outside the kernel, which is different from most of other available algorithms that predict the modules by multiple nodes within the kernel extending to the nodes outside the kernel.

When the algorithm is over, we get the final set of the predicted protein complexes. Subsequently, we need to determine the possibility that two final kernels belong to the same protein complex according to the degree of overlapping. For the protein complexes *i * and *j *, the overlap ratio between them is shown in (8):

(8) $$O_{ij}=\frac{\left|C_{i}\cap C_{j}\right|}{\left|C_{i}\cup C_{j}\right|}$$

Where $C_{i}$ is the number of nodes in cluster *i *, and $C_{j}$ is the number of nodes in cluster *j *. [see Additional file 1 for Algorithm 3--Multistage Kernel Extension (MKE) Algorithm and for its Time Complexity Analysis].

## Results and discussion

For the protein-protein interaction network data of all species, yeast protein-protein interaction network data is relatively complete, so the yeast protein-protein interaction network is selected as the main study object of the experiment. The experiment tests on Krogan dataset [10] and Collins dataset [11] to compare with other algorithms, and then analyses the biological significance of the predicted protein complexes. After removing the self-interactions loop links of the protein nodes and the multilateral links of protein nodes in the pre-process, Krogan dataset and Collins dataset contain 3672, 1622 nodes and 14317, 9074 edges respectively.

Palla et al. (2005) have proposed algorithm CPM (Clique Percolation Method) which can identify the overlapped network cluster structures [12]. The basic hypothesis of the algorithm is: network cluster is made up of multiple adjacent k-cliques, where k-clique is the maximally connected subgraph containing *k * protein nodes. Provided that two k-cliques have k-1common nodes, then the two k-cliques are thought to be adjacent. The CPM algorithm produces the maximally connected subgraph as the module by incessantly uniting adjacent k-cliques. Adamcsek et al. have employed algorithm CPM to develop a network module mining software called CFinder which can expediently dig protein complexes from the protein-protein interaction network. Compared with other graph clustering algorithms, algorithm CPM is a deterministic method, and it can find overlapped protein complexes from the protein-protein interaction network. Algorithm MCL (Markov Clustering) is a fast and scalable unsupervised clustering algorithm, and its basic idea is that: this method first simulates random walk in the graph, then divides the protein-protein interaction network into disjoint dense subgraphs, and finally extracts complexes from the protein-protein interaction network [13]. In the experiment, the maximal size of cliques of the CFinder is set to be 3. The reference protein complexes dataset adopted by algorithm MCL and CFinder comes from reference [14]. And for algorithm MKE, it derives from reference [15].

### Analysing extended level parameter *α *

In order to evaluate the predicted protein complexes, 408 protein complexes are artificially extracted to generate a elaborated catalogue from the published small scale experimental data, and a reference protein complexes dataset is created by filtering out 236 protein complexes with size at least 3, and the average size is 6.7. Meanwhile, since protein complex with size less than 3 is meaningless, the size of all predicted protein complexes analyzed in this paper is at least 3.

Different datasets have different network topology, and the organizational structures of clusters in different datasets vary too. Therefore we need to adjust the extended level parameter *α * of the algorithm to optimize the results of the algorithm on a given dataset.

**Figure 2:**
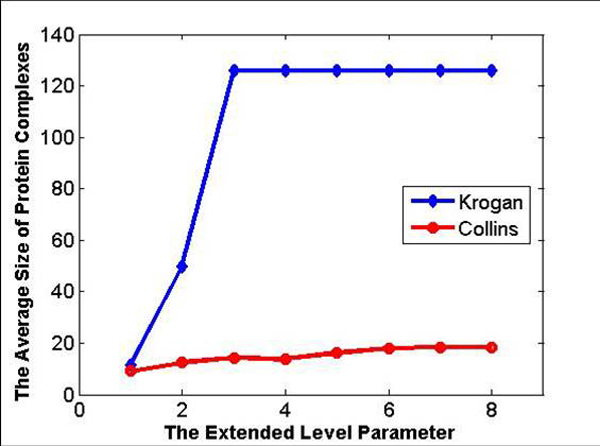
**The average size of complexes predicted under different extended level parameter *α ***. The impact that the extended level parameter *α *. *α * has on the Krogan and Collins dataset.

The MKE algorithm generates the protein complexes by extending the kernel clusters, but different datasets have different optimal extended level parameter *α *. The impact that the extended level parameter *α * has on the Krogan and Collins dataset is shown in Figure 2, the average size of the protein complexes the algorithm discovered on the Krogan dataset dramatically increases with the larger parameter *α *. Although the average size of the protein complexes is stable when parameter *α * is 4, it is far from the average size of the reference protein complex set. On Collins dataset, the average size of the protein complexes basically exhibits the similar trends. From Figure 2, for Krogan and Collins datasets, when α=4 and α=7 respectively, it can be seen that the average size of the protein complexes becomes stable. It indicates that all of kernels in the Krogan and Collins datasets experience at least 4 or 7 times of extension to meet the condition that the increased number of nodes in the next kernel extension is less than that in the previous kernel extension.

### Accuracy of algorithms

The accuracy measure Acc (accuracy) introduced by Brohee and Van Helden is usually used to compare the performance of algorithms [16], which is the geometric mean of the sensitivity Sn and positive predictive value PPV. Sn and PPV are calculated based on matching matrix of predicted protein complexes and reference protein complexes. The number of rows *n * represents the number of reference protein complexes, the number of columns *m * represents the number of predicted protein complexes, the element of the matrix t(i,j)denotes the number of co-occurrence proteins which present in the i-th reference protein complexes and appear in the j-th predicted protein complexes at the same time, and n(i) represents the size of the i-th reference protein complexes. Sn and PPV can be given by

(9) $$Sn=\frac{\sum_{i=1}^{n}\overset{m}{\underset{j=1}{\text{max}}}t\left(i,j\right)}{\sum_{i=1}^{n}n\left(i\right)}$$

(10) $$PPV=\frac{\sum_{j=1}^{m}\overset{n}{\underset{i=1}{\text{max}}}t\left(i,j\right)}{\sum_{j=1}^{m}\sum_{i=1}^{n}t\left(i,j\right)}$$

The accuracy measure of algorithm can be defined as

(11) $$Acc=\sqrt{Sn\times PPV}$$

Respectively calculating these above measures of each algorithm on Krogan and Collins dataset, the results are listed in Table 1. The figures of CFinder and MCL derive from literature [17].

**Table 1:** Various performance indicators of different algorithm on Krogan and Collins datasets

Dataset	Method	clusters	matched	Sn	PPV	Acc
Krogan	CFinder	121	34	0.611	0.162	0.315

	MCL	483	68	0.411	0.408	0.409

	***MKE***	**156**	**82**	**0.384**	**0.522**	**0.448**

***Collins***	***CFinder***	114	66	0.661	0.367	0.492

	***MCL***	183	88	0.587	0.409	0.536

	***MKE***	**115**	**88**	**0.496**	**0.664**	**0.574**

The figures of CFinder and MCL derive from literature [17]

In Table 1 On Krogan dataset, the MKE algorithm separately finds 35 protein complexes and matches 48 protein complexes more than CFinder. Even though the value of Sn of CFinder is 0.227 higher than MKE, the value of PPV of MKE is notably higher than CFinder. With respect to MCL, the predicted clusters is much greater than that MKE predicted, but MKE matches more than that MCL matches, which indicates that in MCL algorithm, multiple predicted protein complexes match one reference protein complex. Just like CFinder, the value of Sn of MCL is 0.027 higher than MKE, but the value of PPV of MKE is 0.114 higher than MCL.

On Collins dataset, the performance of CFinder and MCL exhibits high similarity as on Krogan dataset. Since the values of Sn and PPV of CFinder are extremely uneven, it results in lower values of Acc, which are 0.133 and 0.082 lower than Acc of MKE algorithm respectively on Krogan and Collins dataset. Consequently, on the whole, the MKE algorithm outperforms CFinder. Although Sn and PPV of MCL are relatively balanced on both dataset and there is no significantly difference on the values of Acc between MCL and MKE, MCL is slightly inferior to MKE algorithm.

### Function enrichment analysis

To assess the biological significance of the predicted protein complexes, we can calculate the P-value of the probability that the proteins with common function appear in the predicted protein complexes. P-value reflects the extent to which a given function enrich a protein complex, which is defined as

(12) $$P-value=1-\overset{k-1}{\underset{i=0}{\sum }}\frac{\left(\begin{array}{c} F\\ i \end{array}\right)\left(\begin{array}{c} N-F\\ C-i \end{array}\right)}{\left(\begin{array}{c} N\\ C \end{array}\right)}$$

Let *N * denote the total number of nodes in protein-protein interaction network and C represent the number of proteins within the predicted protein complex, let k and F denote the number of proteins with a given function in protein complex and in PPI network, respectively. If P-value of the predicted protein complex is very low, then it explains that the probability of occurrence of these proteins in the network together exhibiting a given function as a protein complex is very small.

Generally speaking, the function corresponding to the minimal P-value of the predicted protein complex is taken as its primary function or annotation function. Here, we adopt SGD's GO::TermFinder [18] to calculate P-value of the predicted protein complexes in the biological process of GeneOntology, as shown in Table 2. The P-value of these predicted protein complexes found on Krogan and Collins datasets are very low, far smaller than the usual threshold 0.01, which has great biological significance. Among the 10 protein complexes listed from Krogan and Collins datasets, there are 6 predicted protein complexes that can match with reference protein complexes 100%. That is to say, they are the real protein complexes, which firmly prove that MKE can effectively identify the protein complexes with biological significance and even perfectly matched with reference protein complexes.

**Table 2:** The five protein complexes with minimal p-value by MKE mining algorithm

Dataset	ID	P-value	Identification	Gene Ontology term
Krogan	1	4.78e-47	26 out of 26 genes, 100.0%	RNA splicing, via transesterification reactions with bulged adenosine as nucleophile

	2	9.26e-41	15 out of 15 genes, 100.0%	chromatin disassembly

	3	2.47e-30	19 out of 21 genes, 90.5%	mitochondrial translation

	4	7.92e-26	19 out of 23 genes, 82.6%	modification-dependent protein catabolic process

	5	3.80e-19	17 out of 17 genes, 100.0%	transcription from RNA polymerase II promoter

***Collins***	1	1.98e-91	67 out of 93 genes, 72.0%	cytoplasmic translation

	2	1.07e-28	25 out of 25 genes, 100.0%	transcription from RNA polymerase II promoter

	3	1.42e-41	24 out of 24 genes, 100.0%	mitochondrial translation

	4	6.79e-28	14 out of 15 genes, 93.3%	mRNA 3'-end processing

	5	5.17e-44	16 out of 16 genes, 100.0%	chromatin disassembly

### Semantic similarity and co-localization enrichment

Co-localization enrichment analysis is based on the fact that a protein complex can be formed only when its constituents are to be found in the same cellular compartment, and also that protein complex tends to be responsible for a given biological function and a molecular process [19]. For a single protein complex, the co-localization enrichment ratio is the maximum fraction of proteins in the complex which are found at the same localization. For a protein complex set, the co-localization enrichment is the mean co-localization enrichment ratio of all complexes in the set, weighted by the sizes of the complexes, as shown in (13)

(13) $$L=\frac{\sum_{j=1}^{m}\frac{{\text{max}}_{i=1}^{n}l_{i,j}}{N_{j}}}{m}$$

Where, the number of predicted protein complexes and reference cellular compartment protein complexes are respectively *m * and *n *, $N_{j}$ is the size of the predicted protein complex *j *, $l_{i,j}$ is the number of nodes that are found both in reference cellular compartment protein complex *i * and predicted protein complex *j *.

The GO (Gene Ontology) semantic similarity of the protein complex refers to the average degree of association of all protein pairs [20]. The GO semantic similarity of the protein complex set can be obtained by calculating weighted average of all protein complexes. In general, the protein complex with higher GO semantic similarity shows that the probability of proteins within the protein complex expressing the similar function is greater. This paper employs genome co-localization reference dataset compiled by literature [21]. In a protein complex set predicted by a given algorithm, the more protein complexes positioning in the same cellular compartment indicates the stronger recognition capability of the algorithm. This paper adopts the genome co-localization reference dataset from literature [21] and the ProCope [22] tool to analyze the GO semantic similarity and co-localization enrichment on the results predicted by each algorithm on Krogan and Collins dataset.

In the Table 3 the figures of Reference dataset derived from literature [21], on Krogan dataset, the GO semantic similarity and Co-localization scores of CFinder are 0.144 and 0.125 lower than that of MKE respectively, so it is natural that the arithmetic mean of MKE is much greater than that of CFinder. Compared to MCL, the co-localization enrichment of MKE is 0.109 lower than that of MCL, but the GO semantic similarity of the MKE algorithm is 0.197 higher than that of MCL. Finally, by calculating the arithmetic mean of these two metrics, we find that the result of MKE is 0.044 higher than that of MCL. On Collins dataset, CFinder algorithm behaves with high consistency as on Krogan dataset. However, the GO semantic similarity of MCL is 0.042 lower than that of MKE, whereas the Co-localization score of MCL is 0.201 higher than that MKE calculated. The latter indicator produces a significant difference that leads to the lower arithmetic mean of MKE compared to MCL. In view of this outcome, we make a further analysis. As shown in Table 3 the indicators calculated by each algorithm on Collins dataset are all increased when compared to the Krogan dataset, which proves the specificity of different networks. MCL performs better on Collins dataset than on Krogan dataset, indicating that one algorithm is not applicable to all kinds of networks. Moreover, referring to Table I, MCL finds much more clusters than that MKE predicted which may be an advantage when calculating the Co-localization score.

**Table 3:** GO semantic similarity and Co-localization enrichment analysis by algorithm MKE

Dataset	Method	GO semantic similarity score	Co-localization score	Arithmetic mean
Krogan	**CFinder**	0.482	0.448	0.465

	**MCL**	0.429	0.682	0.556

	**MKE**	**0.626**	**0.573**	**0.600**

***Collins***	**CFinder**	0.725	0.616	0.671

	**MCL**	0.783	0.900	0.842

	**MKE**	**0.825**	**0.699**	**0.762**

***Reference***		0.984	0.768	0.876

The figures of Reference dataset come from literature [21]

Therefore, by and large, although MKE algorithm is not better than all of the selected algorithms, MKE performs better than algorithm CFinder on the aspects of GO semantic similarity and co-localization enrichment, and can effectively detect the protein complexes with biological significance in the protein-protein interaction network.

## Conclusion

Due to the complexity of structure and the limitations of the experimental validation of the protein-protein interaction network, there is no convincing and strict definition regarding the verification standard of the protein complex up to now. Therefore, for protein complex mining, the detecting standard of the protein complex should be first confirmed. That is to say, what is the structure of the protein complex needs to be defined.

The formation of the protein-protein interaction network follows its intrinsic law and the PPI network gradually develops by some protein complexes with inherent links. The criticality is an important property of the protein complex. Critical proteins can always be discovered within protein complexes, which are the high-degree nodes with many adjacent nodes. The protein nodes with higher degree tend to exhibit the criticality on biological properties as kernels and play an important role in the protein complex. Thus one or multiple critical nodes can be taken as kernels around which there are a lot of adjacent protein nodes closely connecting with each other, and the periphery of these adjacent proteins have also some adjacent nodes. All these nodes construct a relatively independent set which is able to implement some relatively independent biological functions. In other words, such protein sets are most likely to construct protein complexes.

Inspired by the community formation law of the complex social network and the centrality-lethality rule, and combining the idea of critical protein nodes detection, this paper proposes a new protein complex mining algorithm MKE based on multistage kernel extension. MKE is the first algorithm to identify the innermost kernel of the protein complex, namely taking the critical nodes with high degree and more adjacent nodes as the first level kernel of the protein complex. Then MKE expands the first level kernel to be the second level kernel of the protein complex by adding the weighted best neighbour node into the current kernel, and repeatedly goes on expansion stage-by-stage to construct protein complex, and then MKE merges overlapped protein complexes to form the protein complex set. MKE has better accuracy compared with the classical clique percolation method and markov clustering algorithm. MKE also performs better than classical clique percolation method both on Gene Ontology semantic similarity and co-localization enrichment and can effectively identify protein complexes with biological significance in the PPI network.

## Competing interests

The authors declare that they have no competing interests.

## Authors' contributions

XS designed the protein complexes mining algorithm based on multistage kernel extension and weighted best neighbour node. YL implemented the protein complexes mining algorithm and run the experiments. YZ and JY helped plan the experiments analysed and contributed to writing the manuscript. TH and XTH supervised and helped conceive the study. All authors read and approved the final manuscript.

## Supplementary Material

Additional file 1 **Supplementary Algorithms and Time Complexity Analysis**. A collection of algorithms and their corresponding time complexity analysis is available in Additional_file_1.pdf. Format: PDF. size: 123KB Click here for file

## Declarations

Publication of this article has been funded by NSF IIP 1160960, NSF IIP 1332024.

This article has been published as part of *BMC Bioinformatics *Volume 15 Supplement 12, 2014: Selected articles from the IEEE International Conference on Bioinformatics and Biomedicine (BIBM 2013): Bioinformatics. The full contents of the supplement are available online at http://www.biomedcentral.com/bmcbioinformatics/supplements/15/S12.
