# Supplementary material for: An efficient protein complex mining algorithm based on Multistage Kernel Extension
Source: BMC Bioinformatics. 2014 Nov 6;15(Suppl 12):S7. doi: 10.1186/1471-2105-15-S12-S7 (PMC4255745; doi:10.1186/1471-2105-15-S12-S7)
Supplement: Additional file 1 — Supplementary Algorithms and Time Complexity Analysis. A collection of algorithms and their corresponding time complexity analysis is available in Additional_file_1.pdf. Format: PDF. size: 123KB [file 1471-2105-15-S12-S7-S1.pdf]

Supplementary Algorithms and Time Complexity Analysis

Algorithm 1: Identifying the First Level Kernel (IFLK) Algorithm

Input: Node set of graph as V

Output: First level kernel set C.

1 Find the proper k value to make $P(k) \le 0.01$.

2 ***for*** each node $v \in V$

3 ***if*** $d_v \ge k$

4 $H = H \cup \{v\}$ ·

5 ***Endif***

6 ***Endfor***

7 ***if*** $H \in \phi$

8 $H \leftarrow v$ (*with the largest degree*)

9 ***Endif***

10 ***for*** each node $s \in H$

11 $C = \{s\}$, and marks node s.

12 ***for*** each unmarked node $t \in H$ and $t \ne s$

13 ***if*** $w_{st} \ge w_{ave}$ ***and*** $w_{ts} \ge w_{ave}$

14 $C = C \cup \{t\}$.

15 ***Endif***

16 ***Endfor***

17 ***Endfor***

18 output all the clusters as first level kernel set C.

In the IFLK algorithm, V is the set of nodes in PPI network, v is a node in the set V. H is the set of initial kernel nodes of the first level kernel of protein complexes. w_{ave} is the initial threshold of the weight, initialized to 0.8 in this paper. s and t are the nodes in set H. C represents the set of the first level kernels of protein complexes.

Since the extent of the closeness of interactions within the protein complexes are not uniform in the PPI, only part of the first level kernels of protein complexes with higher connection density can be obtained. If the number of nodes in the network is n, and $P(k)=0.01$, then from step 1 to step 9, the time complexity is $O(n/P(k))$, and from step 10 to step 17, the time complexity is still $O(n/P(k))$, therefore, the total time complexity of IFLK algorithm is $O(n/P(k))$.

Algorithm 2: Identifying the Second Level Kernel (ISLK) Algorithm

Input: First level kernels of protein complexes as f.

Output: Second level kernels of protein complexes.

1 Find all the direct neighbor nodes of f as N_f.
2 Calculate the average weight of subnetwork of f and N_f as w_{ave}.
3 ***for*** each node $v \in N_f$
4 find its best neighbor $Bn(v) \in f$
5 ***if*** $w_{v,Bn(v)} < w_{ave} \,||\, w_{Bn(v),v} < w_{ave}$
6 $N_f = N_f - \{v\}$.
7 ***Endif***
8 ***Endfor***
9 Output f and N_f as the second level kernels of protein complexes.

In the ISLK algorithm, let n denote the number of nodes within the network and n_c represent the size of the next level kernel extended. The first step traverses all the nodes to find the neighbour node set of the current cluster, so the time complexity is $\mathrm{O}(n)$. The second step demands a weighted superposition of all pairs of nodes of the subgraph to compute the average weight, thus the time complexity is $\mathrm{O}(n_c^2)$. Step 3 to step 8 are to determine the relationship between the nodes in the set N_f and nodes in the set f, thus the time complexity is $\mathrm{O}(n_c^2)$. Commonly speaking, $n_c \ll n$, so the whole time complexity of ISLC algorithm is far less than $\mathrm{O}(n^2)$.

Algorithm 3: Multistage Kernel Extension (MKE)Algorithm

Input: undirected and unweighted graph $G(V,E)$.

Output: all the protein complexes of PPI network.

Transform the undirected and unweighted graph G into directed and weighted graph G''.
Repeat
Call Algorithm IFLK to generate the first level kernel of protein complex noted as f.
Repeat
for each current level kernel as cc
call Algorithm ISLK to generate its next level kernel as nc.
mark all nodes of kernel nc in PPI network.
Endfor
Until $\Delta N_{current} \le \Delta N_{prior}$ ***and*** $\alpha > T_\alpha$
Until no unmarked node left in PPI network.
for each two final level kernels p and q
find the maximal overlapped clusters m and n.
If $O(m,n) \ge 0.5$
$m = m \cup n$; $n = \phi$.
End if

16 ***Endfor***

17 Output the final cluster set as protein complexes.

In the MKE algorithm, given n represents the number of nodes in the network, T_α is a given threshold value of Extended Level Parameter, $d_{\max}$ denotes the maximum degree of nodes in the network, μ is the number of the protein complex kernels before merging, K is the extended progression, ΔN is the reduced number of protein complex kernels in the process of merging. The first step is to transform the undirected graph to directed and weighted graph, so the time complexity is $\mathrm{O}(n(d_{\max})^2)$. For IFLK algorithm in the third step and ISLK algorithm in the six step, the time complexity are $O(n/P(k))$ and $O(n)$ separately. From step 2 to step 10, the time complexity is determined by the extended progression and the number of protein complexes generated, therefore, the time complexity is $\mathrm{O}(\mu \bullet K \bullet n)$. For the step 12, it demands calculation on all pairs of protein complexes to find the pair of protein complexes with largest degree of overlapping, thus the time complexity is $\mathrm{O}(\mu^2)$. Then, from the step 11 to step 16, the time complexity is $\mathrm{O}(\mu^2 \Delta N)$ that MKE algorithm merges the overlapped protein complexes. Since $\mu \Delta N$ is far less than n , the total time complexity of MKE algorithm is $\mathrm{O}(n(d_{\max})^2+\mu Kn+\mu^2 \Delta N)$, namely $\mathrm{O}(n(d_{\max})^2+\mu Kn)$.
